# Striatal volume is related to phonemic verbal fluency but not to semantic or alternating verbal fluency in early Parkinson’s disease

**DOI:** 10.1007/s00702-013-1073-2

**Published:** 2013-08-03

**Authors:** Ulla Ellfolk, Juho Joutsa, Juha O. Rinne, Riitta Parkkola, Pekka Jokinen, Mira Karrasch

**Affiliations:** 1 Department of Psychology and Logopedics, Abo Akademi University, 20500 Turku, Finland; 2 Division of Clinical Neurosciences, Turku University Hospital, University of Turku, Turku, Finland; 3 Turku PET Centre, Turku University Hospital, University of Turku, Turku, Finland; 4 Department of Radiology, Turku University Hospital, University of Turku, Turku, Finland; 5 Department of Radiology, Tampere University Hospital, University of Tampere, Tampere, Finland

**Keywords:** Early stage Parkinson’s disease, Voxel-based morphometry, Verbal fluency, Semantic fluency, Phonemic fluency, Alternating fluency

## Abstract

Verbal fluency impairments are frequent in Parkinson’s disease (PD) and they may be present already at early stages. Semantic fluency impairment is associated with Parkinson’s disease dementia and temporal, frontal and cerebellar cortical changes. Few studies have addressed cerebral structural correlates of different verbal fluency tasks in early stage PD. We therefore studied gray matter volumes of T1-weighted MRI images using voxel-based morphometry in relation to semantic, phonemic, and alternating verbal fluency in younger (mean age <65 years), early stage (mean disease duration <3 years), non-demented PD patients (*n* = 28) and healthy controls (*n* = 27). We found a significant association between worse phonemic fluency and smaller striatal, namely right caudate gray matter volume in the PD group only (family-wise error corrected *p* = 0.007). Reduced semantic fluency was associated with smaller gray matter volumes in left parietal cortex (*p* = 0.037) and at trend level with smaller bilateral cerebellum gray matter volume across groups (*p* = 0.062), but not in the separate PD or control groups. There were no significant relationships between alternating fluency and gray matter volumes in the whole sample or in the groups separately. The fact that phonemic fluency, but not semantic or alternating fluency, was associated with caudate gray matter volume at early stage PD suggests that different fluency tasks rely on different neural substrates, and that language networks supporting semantic search and verbal-semantic switching are unrelated to brain gray matter volume at early disease stages in PD.

## Introduction

Verbal fluency impairments are among the earliest and most common cognitive deficits in Parkinson’s disease (PD) (Henry and Crawford 2004a; Muslimovic et al. 2005), and they are related to incipient dementia (PDD) (Williams-Gray et al. 2009; Evans et al. 2011). Semantic fluency is generally more impaired than phonemic fluency in PD (Henry and Crawford 2004a; Koerts et al. 2013), although some studies with non-demented PD patients have reported a reversed pattern (Ibarretxe-Bilbao et al. 2011), or even no differences in relation to healthy controls (Tröster et al. 1998; Piatt et al. 1999; Fine et al. 2011; Herrera et al. 2012). Early stage and non-demented PD patients may be impaired on more demanding types of fluency, such as proper name fluency (Fine et al. 2011) or alternating fluency (Zec 1999). In relation to semantic and phonemic fluency, alternating fluency has been reported to be most strongly affected in non-demented PD (Zec 1999).

Both semantic fluency (words belonging to a semantic category, often animals) and phonemic fluency (words beginning with a specific letter) are dependent on extensive cortical and subcortical networks, with the most significant area being the left inferior frontal cortex (Hirshorn and Thompson-Schill 2006; Costafreda et al. 2006). Moreover, semantic and phonemic fluency are believed to be dependent on partially different neural circuits, with semantic fluency relying on temporal areas and phonemic fluency on more frontal regions (Henry and Crawford 2004b). A reversible impairment in phonemic fluency, but not semantic fluency, has been demonstrated in non-demented, dopamine deprived PD patients (Herrera et al. 2012), supporting the assumption that semantic and phonemic fluency rely on distinct neural connections. Structural findings from voxel-based morphometry (VBM) studies in healthy individuals also suggest a role of the caudate in verbal fluency, which is more pronounced for phonemic than for semantic fluency (Grogan et al. 2009). Alternating verbal fluency (switching between semantic categories or letters) has been found to be subserved by the left inferior frontal gyrus (Hirshorn and Thompson-Schill 2006) and regions in the posterior parietal cortex (Gurd 2002), bilateral premotor areas, superior parietal cortex, ventral occipito-temporal cortex and posterior cingulate areas (Birn et al. 2010). Morphometric evidence also suggests a role of putamen volume in verbal switching (Thames et al. 2012).

Cerebral structural correlates of verbal fluency in PD have been sparsely investigated using VBM. One previous study (Pereira et al. 2009) with non-demented PD patients found that impaired semantic fluency was related to temporal, frontal, and cerebellar gray matter (GM) reduction, whereas impaired phonemic fluency scores were not related to GM volumes. A recent study (Pagonabarraga et al. 2013) assessing cortical thickness found an association between reduced alternating verbal fluency and cortical thinning in right parahippocampal gyrus, left lingual gyrus and left precuneus. This association was present in PDD patients, but not in cognitively normal or PD-MCI (mild cognitive impairment) patients. Both of the above-mentioned studies included older (mean age >65 years) non-demented PD patients with disease durations more than 10 years. To date, no structural neuroimaging studies comparing different types of verbal fluency in younger PD patients with shorter disease duration have been conducted.

The aim of the present study was to examine brain GM correlates of semantic, phonemic, and alternating verbal fluency in early stage, non-demented PD patients and healthy controls using VBM. Semantic and phonemic word fluency tasks are widely used simple and time-efficient clinical measures of frontal–temporal cortical functioning. Alternating fluency tasks provide additional frontal-executive cognitive demand as they require both switching between two sematic categories and inhibiting category-unrelated responses. Executive functioning, including impaired set-shifting ability, is considered a core cognitive deficit in PD (Brown and Marsden 1990; Owen et al. 1992).

## Participants and methods

### Participants and clinical assessment

The study sample has been reported previously (Ellfolk et al. 2012). In summary, 28 PD patients and 28 healthy controls were recruited through a national survey research project on PD cognition in collaboration with the Finnish Parkinson Association and from two outpatient neurology clinics. Inclusion criteria were short PD duration (<3 years) and exclusion criteria were dementia, major depression, any neurological or psychiatric disease, major vascular lesions, and traumatic brain injury. All patients were diagnosed with idiopathic PD according to UK Brain Bank criteria by their treating neurologist, none were taking anticholinergic medication, and none had structural brain findings inconsistent with the PD diagnosis. Motor impairment was rated using the validated Finnish version of the Unified PD Rating Scale (UPDRS-Fin III, http://www.parkinson.fi/sites/default/files/UPDRS-FIN%20_III.pdf). Exclusion of dementia was done according to Movement Disorder Society Task Force recommendations (Emre et al. 2007; Dubois et al. 2007). Based on clinical impression, a semi-structured interview and formal neuropsychological assessment, none had cognitive deficits significantly impairing daily life. All patients were functionally independent.

The group of healthy controls consisted of 15 men and 13 women. None of them were clinically diagnosed with depression, neurological or psychiatric disease, or had a history of head trauma. Based on visual evaluation of MRI images, one control subject was diagnosed with asymptomatic meningeoma, and was therefore not included in the VBM analyses. Patients and controls were equated for age and years of education on group level (for information, see Table 1).

**Table 1:** Demographic, clinical, and verbal fluency comparisons between PD patients and controls

	PD (*n* = 28)	Controls (*n* = 28)	*p* value
Demographic and clinical^a^			
Gender (male/female)^b^	14/14	15/13	NS
Age	60.3 (8.1)	61.3 (7.2)	NS
Education (years)	13.8 (3.5)	14.8 (3.2)	NS
MMSE score	28.0 (2.0)	28.1 (1.9)	NS
Disease duration (months)	18.9 (10.8)	NA	
Age at PD onset	58.8 (7.8)	NA	
UPDRS-III score	25.9 (7.5)	NA	
Levodopa equivalent dose (mg/day)	392.0 (230.3)	NA	
Total intracranial volume (TIV) (ml)^c^	1,515.1 (155.8)	1,482.3 (160.8)	NS
Verbal fluency performances^d, e^			
Semantic fluency (animals)	24.8 (6.3)	26.6 (5.3)	NS
Phonemic fluency (letter S)	14.5 (5.8)	16.4 (5.0)	NS
Alternating fluency (animals, furniture)	15.3 (3.9)	17.3 (2.8)	0.028

^a^Independent samples *t* test

^b^Pearson Chi-square test

^c^TIVs obtained from native-space images, control *n* = 27

^d^Univariate ANOVA

^e^Total correct words during 60s

The PD patients were neurologically evaluated by experienced clinical investigators at the Turku PET Centre (JJ and PJ). Brain MRI scans were interpreted by an experienced neuroradiologist (RP). Neuropsychological assessments and interviews were performed by an experienced clinical neuropsychologist (UE) and a trained assistant at the Department of Psychology and Logopedics at Abo Akademi University. All participants gave written consent. The study protocol was approved by the Joint Ethics Committee of Turku University and Turku University Hospital.

### Verbal fluency measures

Semantic fluency was assessed by the generation of animal names during 60s. Phonemic fluency was assessed by the generation of words beginning with the letter S during 60s. Alternating fluency was assessed according to the Parkinson neuropsychometric dementia assessment (PANDA) (Kalbe et al. 2008). The participant was asked to alternate between two semantic categories, animals and furniture. Both the number of correct words and switching errors were registered. Group differences on the verbal fluency measures were assessed using univariate ANOVA and ANCOVA using SPSS 21. Associations between verbal fluency, clinical and demographic background variables were examined using correlation analyses. The total correct raw scores of the three fluency tasks were examined in relation to brain GM volume using VBM.

### MRI

A 1.5-T scanner (Philips Gyroscan Intera 1.5T CV Novo Dual, Philips Healthcare, Best, the Netherlands) equipped with a SENSE head coil was used to obtain T1- and T2-weighted, diffusion and flair sequences. T1-weighed three-dimensional fast field echo images were obtained in transverse planes with contiguous 1 × 1 × 1 mm voxels, 25 ms echo time, 30° flip angle, and field-of-view 256 × 256 mm yielding at least 160 contiguous slices.

The preprocessing of the T1-weighted images were performed with the VBM8 toolbox for SPM (Christian Gaser, University of Jena; http://dbm.neuro.uni-jena.de/vbm8/) and the voxel-wise analyses of the gray matter segments were conducted using SPM8 (Wellcome Department of Cognitive Neurology, London, UK) running in Matlab R2011a (MathWorks, Natick, MA). Total intracranial volumes (TIVs) were obtained from native-space images. The normalization to MNI space was achieved using a high-dimensional DARTEL normalization procedure (Ashburner 2007). The analyses were conducted using modulated images (1.5 × 1.5 × 1.5 mm voxels) (Ashburner and Friston 2000), which were smoothed with a 10-mm full width at half maximum (FWHM) Gaussian kernel. The images were thresholded with voxel value 0.1 to include the whole brain and restrict the analyses only to the gray matter.

Potential confounding factors (gender, age, handedness, education and TIV in the whole sample; and UPDRS score in PD patients) were analyzed using single variable regression. Age and TIV were associated with local GM volumes and thus included to the multiple regression analyses as nuisance covariates. Also sex had an effect on the local GM volumes, but was not included as a covariate, because of a strong intercorrelation with TIV as men had larger TIV compared to women. The variables of interest included semantic, phonemic, and alternating fluency. The analyses were performed at height threshold of uncorrected *p* < 0.005, and family-wise error (FWE) corrected cluster-level *p*_fwe_ < 0.05 was considered statistically significant. The results were visualized by using Mango (version 2.6, http://ric.uthscsa.edu/mango/).

## Results

### Verbal fluency performances, demographic and clinical background variables

PD patients and controls did not differ regarding gender, age, education, MMSE (Mini-Mental State Examination) scores, or TIVs. The PD patients performed significantly worse than controls on the alternating fluency task, but they performed equal to controls on the semantic and phonemic fluency tasks. The analyses were re-run with age and education as covariates, resulting in the same significance pattern. Details are shown in Table 1. Inspection of the alternating fluency data revealed that six PD patients made 1–4 shifting errors, whereas in the control group, only one participant made an error on the alternating fluency task.

In the PD group, a correlation analysis revealed that worse alternating fluency score was significantly associated with older age, *r* = −0.47, *p* = 0.013, and lower MMSE score, *r* = 0.42, *p* = 0.025. A trend, *r* = 0.37, *p* = 0.050, toward a relationship between worse semantic fluency and lower MMSE score was also present in the PD group. None of the verbal fluency measures were associated with UPDRS-III score or education in the PD group. In controls, there was a significant positive association between phonemic fluency, *r* = 0.59, *p* = 0.001, and education, as well as alternating fluency and education, *r* = 0.40, *p* = 0.034. No significant associations between any of the verbal fluency tasks and age or MMSE scores were present in controls.

### VBM

**Fig. 1:**
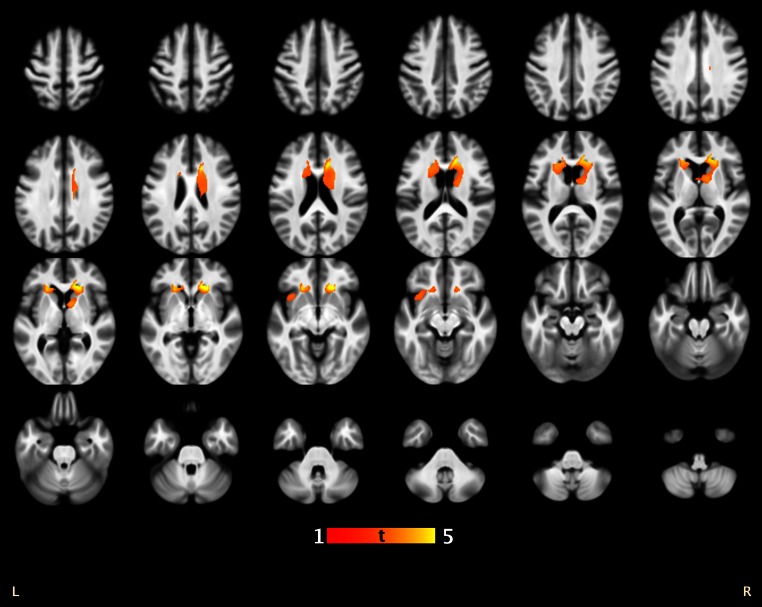
Association between phonemic fluency and brain gray matter volume in Parkinson’s disease patients. The results are shown at height threshold *T* = 2.80, *p* < 0.005 overlaid on the average normalized T1-weighted image of the whole study group. Only (near) significant clusters are shown

In the PD group, lower phonemic fluency scores were associated with smaller striatal GM volume [cluster size *k* = 3,584 voxels, *p*_fwe_ = 0.007, MNI coordinates (mm) *xyz* = 21 27 −6 (right), *k* = 1,755, *p*_fwe_ = 0.116, *xyz* = −20 24 −9 (left)] (Fig. 1). This association was absent in the control subjects (*p*_fwe_ > 0.99). The significantly associated GM coordinate regions were the right caudate nucleus, with the largest cluster size in regions of the head of the caudate, extending to the caudate body. Associated GM regions in the left hemisphere not reaching statistical significance were mainly the putamen and the left caudate head.

**Fig. 2:**
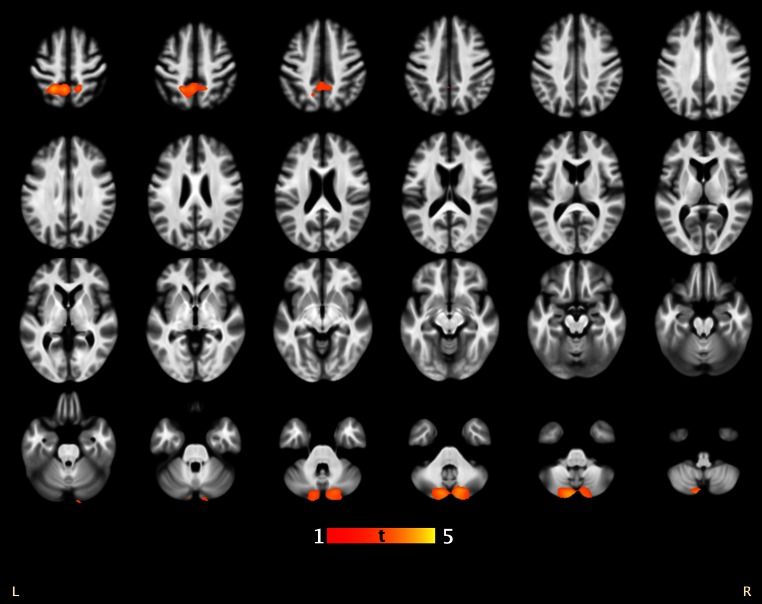
Association between semantic fluency and brain gray matter volume in the whole study group. The results are shown at height threshold *T* = 2.68, *p* < 0.005 overlaid on the average normalized T1-weighted image of the whole study group. Only (near) significant clusters are shown

Additionally, lower scores on the semantic fluency task were associated with smaller local GM volume in left parietal cortex over the whole study group (*k* = 2,759, *p*_fwe_ = 0.034, *xyz* = −20 −49 55) (Fig. 2), but not in groups separately. Associated main regions were located in the left precuneus (Brodmann area 7). A trend-level association was also present between worse semantic fluency and smaller bilateral cerebellar volume over the whole study group (*k* = 2,349, *p*_fwe_ = 0.062, *xyz* = −6 −84 −44). There were no associations between local GM volumes and the alternating fluency task in the whole sample or in the groups separately.

## Discussion

Despite substantial evidence on verbal fluency deficits at different stages of PD (Henry and Crawford 2004a), information about cortical and subcortical volume in relation to different fluency tasks at early stage PD has been scarce. Reduced semantic fluency is related to PD dementia (Williams-Gray et al. 2007) and widespread cortical GM changes (Duncan et al. 2013). We therefore studied brain volumetric correlates of semantic, phonemic and alternating fluency in early stage, non-demented, medicated PD patients and healthy controls.

Our main finding was a significant association between worse phonemic fluency performance and smaller GM volume of the right caudate. This association was present in PD patients, but not in controls. A second finding was that over the whole sample, worse semantic fluency performance was associated with smaller, dominantly left hemisphere parietal GM volume. A trend-level association between worse semantic fluency and smaller bilateral cerebellar GM volume was present over the whole sample, but not in the separate groups. Alternating fluency was not associated with local GM volume in any of the two groups or in the whole sample.

In contrast to the findings of Pereira et al. (2009) who found associations between semantic fluency and temporal, frontal and cerebellar volumes in older, non-demented PD patients, we found a significant association between phonemic fluency, but not semantic fluency, and local brain GM volume in PD patients. This difference may be related to fact that the patients in the present study were younger (mean age <65 years) and were at an earlier disease stage (disease duration <3 years) than the PD patients in the study of Pereira et al. (mean age >70, mean disease duration >10 years), and that our patients had no significant local atrophy when compared to controls. In the present study, we also applied a more conservative statistical threshold, which may partly explain some of the differences between previous and present findings. Our main finding suggests that phonemic processing in early PD is related to striatal volume, mainly the caudate head, in the absence of significant behavioral decline or significant local GM atrophy. Cortico-striatal projections are affected by dopamine depletion in PD (Jellinger 2012), and it has been demonstrated that phonemic fluency deficits, but not semantic fluency deficits in PD, can be modulated by levodopa treatment (Herrera et al. 2012). In the present study, the phonemic fluency and GM volume correlation in the PD group was significant in the right caudate, whereas the association with the left caudate showed only a trend toward significance at corrected threshold levels. Whilst activation studies mainly report language-related tasks in left hemisphere locations (Birn et al. 2010), right caudate dominance for phonemic fluency has been reported in a VBM study in healthy adults (Grogan et al. 2009). Both right (Burton et al. 2004; Apostolova et al. 2010) and bilateral (Nagano-Saito et al. 2005) caudate atrophy have been demonstrated in PDD patients, but it remains unclear how caudate volume loss contributes to cognitive decline in PD. A recent study (Pitcher et al. 2012) reported caudate volume loss in cognitively normal PD, PD-MCI, and PDD patients, but the degree of atrophy was not related to cognitive status. Volumetric GM associations with single cognitive measures such as verbal fluency were not reported.

We found no significant relationship between semantic fluency and GM volume in either the PD group or in controls. However, over the whole group, a significant relationship between worse semantic fluency scores and smaller, dominantly left hemisphere parietal GM volume, mainly including portions of the precuneus, was present. In healthy populations, the precuneus has been associated with a wide spectrum of highly integrated tasks, such as visual imagery and episodic memory retrieval (Cavanna and Trimble 2006). Additionally, over the whole group, there was a trend toward an association between worse semantic fluency and smaller cerebellar volume. The cerebellum has repeatedly been associated with verbal fluency tasks in functional imaging studies (Schlosser et al. 1998; Weiss et al. 2003; Gauthier et al. 2009), and greater cerebellar activation has been demonstrated in easy relative to more demanding fluency task conditions (Senhorini et al. 2011).

Only alternating fluency was significantly impaired in the present PD sample. The alternating fluency task employed in this study required switching between two semantic categories. The fact that the PD patients performed worse than controls on this theoretically most demanding “frontal” task supports previous evidence of larger deficits in alternating semantic fluency than on standard semantic fluency tasks in PD (Zec 1999; Henry and Crawford 2004a; Pagonabarraga et al. 2008). As the alternating fluency task was not related to brain GM volume in the present study, it could be proposed that alternating fluency deficits in early stage, non-demented PD are related to functional, rather than structural, disruptions of the fronto-striatal circuitry, and may best be captured using functional imaging approaches. Associations between attentional set-shifting, dopamine levels and fronto-parietal networks (Williams-Gray et al. 2008) including hypometabolism in the dorsolateral prefrontal cortex (Sawada et al. 2012) have previously been demonstrated in PD. In PDD patients with longer disease duration, alternating fluency has been associated with cortical thinning of the parahippocampal gyrus, lingual gyrus, and precuneus (Pagonabarraga et al. 2013). Based on our behavioral findings, the alternating fluency task was difficult enough to differentiate between cognitively preserved, early stage PD patients and controls, supporting the notion that alternating fluency tasks are useful in early clinical diagnostic settings (Pagonabarraga et al. 2008).

In contrast to the findings of Pereira et al. (2009), we found no associations between verbal fluency performances and degree of motor impairment in the PD group. Most previous PD studies have examined only phonemic and semantic fluency, and reported more pronounced semantic than phonemic fluency deficits (Henry and Crawford 2004a). In healthy elderly, age has been found to have a greater impact on semantic fluency than on phonemic fluency (Mathuranath et al. 2003; Stokholm et al. 2013), suggesting that age is an important factor to consider when comparing verbal fluency findings across studies. It is possible that some of the variability in semantic fluency tasks previously reported in PD may be moderated by the effect of age, as most have studied older (>65 years) PD patients.

In conclusion, we found that phonemic fluency, but not semantic or alternating fluency, was related to brain GM volume in early stage, non-demented PD. Associated regions were striatal, namely portions of the right caudate. Worse semantic fluency was related to smaller left parietal and at trend level bilateral cerebellar, GM volume over the whole group. On the behavioral level, PD patients performed worse than controls only on the alternating fluency task. The results indicate that different verbal fluency tasks are related to different neural substrates, and that alternating fluency is more impaired than semantic and phonemic fluency at early stage, non-demented PD.
